# Metagenomic sequencing and reconstruction of 82 microbial genomes from barley seed communities

**DOI:** 10.1038/s41597-024-03332-x

**Published:** 2024-05-10

**Authors:** Kalonji A. Tshisekedi, Pieter De Maayer, Angela Botes

**Affiliations:** School of Molecular and Cell Biology, Wits University, Johannesburg, South Africa

**Keywords:** Computational biology and bioinformatics, Environmental sciences

## Abstract

Barley (*Hordeum vulgare*) is essential to global food systems and the brewing industry. Its physiological traits and microbial communities determine malt quality. Although microbes influence barley from seed health to fermentation, there is a gap in metagenomic insights during seed storage. Crucially, elucidating the changes in microbial composition associated with barley seeds is imperative for understanding how these fluctuations can impact seed health and ultimately, influence both agricultural yield and quality of barley-derived products. Whole metagenomes were sequenced from eight barley seed samples obtained at different storage time points from harvest to nine months. After binning, 82 metagenome-assembled genomes (MAGs) belonging to 26 distinct bacterial genera were assembled, with a substantial proportion of potential novel species. Most of our MAG dataset (61%) showed over 90% genome completeness. This pioneering barley seed microbial genome retrieval provides insights into species diversity and structure, laying the groundwork for understanding barley seed microbiome interactions at the genome level.

## Background & Summary

Seed microbiomes are essential to plant health, growth, and resilience, and play an important role in the physiological processes required for effective crop development^1^. The barley seed microbiome, in particular, is of critical importance, influencing not only crop yield but also the quality of barley-derived products^2,3^. Barley (*Hordeum vulgare*) has been integral to agriculture since the early phases of human civilization^4^. Its significance in the modern era is two-fold: as a fundamental component of the global food system, and as a crucial ingredient in the brewing industry^3,5^. While the physiological attributes of barley influence malt quality, the microbial communities associated with barley also play an essential role, from sowing to malting^2^.

Malting barley seeds are colonised by rich and diverse microbial communities, encompassing both endophytic and epiphytic organisms^1,6,7^. These microorganisms, which can be both beneficial and detrimental, have the potential to affect seed health, germination success, and the quality of fermentation products^8–10^. Several studies highlight the diversity of microbial populations associated with malting barley and their potential effects on brewing product quality^8,11,12^. Understanding these microbial communities and their genomic content can provide insights into seed storage longevity, contamination risks, and their potential impact on subsequent production stages. However, there is a notable gap in comprehensive metagenomic datasets focusing on these microbial communities, especially during the seed storage phase.

Metagenome sequencing can provide profound insights into microbial ecosystems without necessitating laboratory cultivation^13–15^. This approach not only provides a comprehensive understanding of the taxonomic and functional variations among phytomicrobial communities, but also sheds light on the complex interrelationships across these communities and their plant hosts^16,17^. In the context of barley seed storage, acquiring this understanding using omics paves the way for developing microbial management strategies, optimising storage conditions, mitigating losses, and ensuring consistent production of premium malt.

**Fig. 1:**
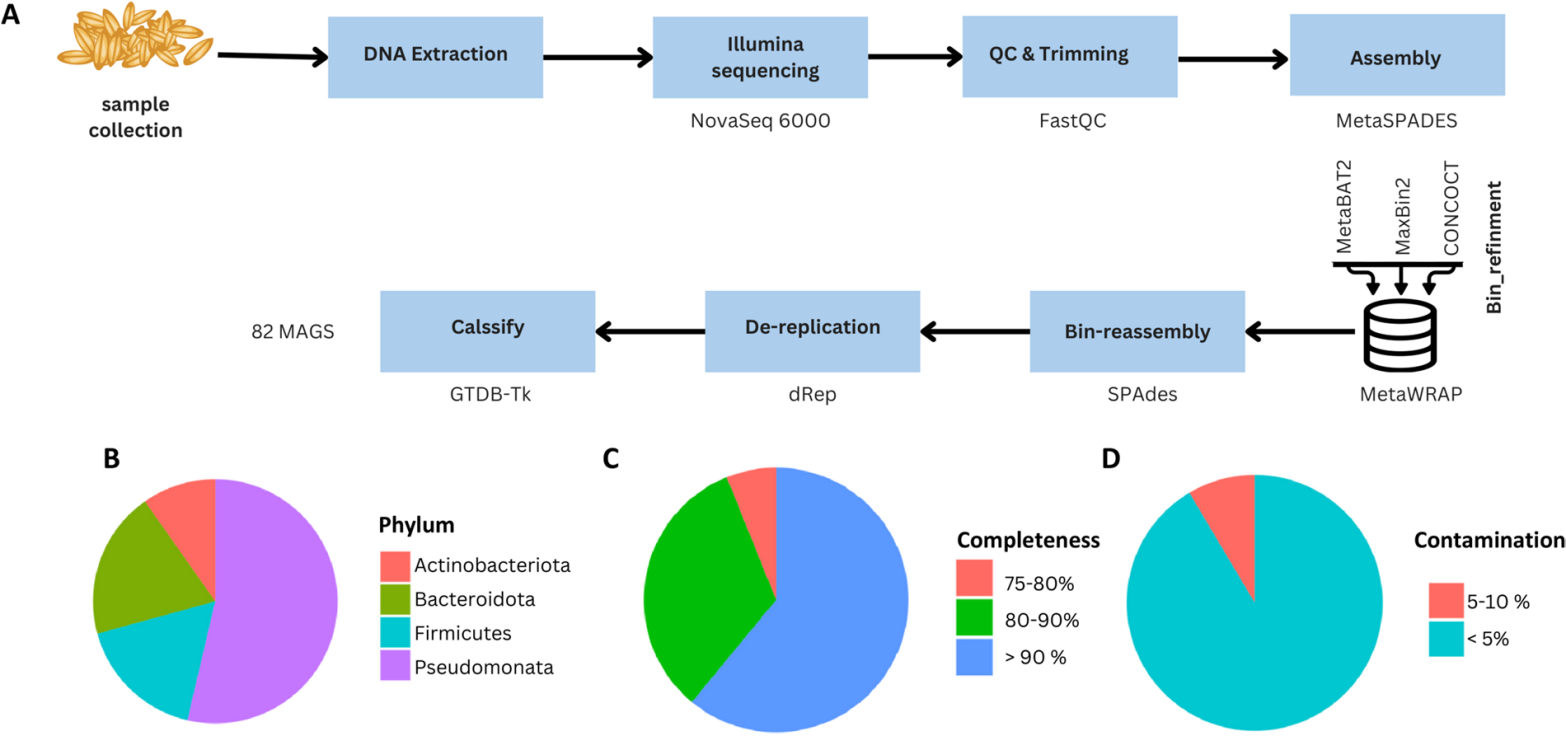
Comparative analysis of phylum distribution, MAGs completeness, and contamination.

Whole metagenomes were sequenced from eight samples of barley seeds stored in siloes at four different time points (two samples per time point), namely at harvest and after three, six and nine months, respectively (Table S1). The metagenomic data was assembled into nearly complete microbial genomes. A total of 82 metagenome-assembled genomes (MAGs) were assembled from these metagenomes (Table S2). The completeness of the MAGs was evaluated using CheckM v1.2.2^18^. All MAGs demonstrated completeness >75%, with 50/82 being >90% complete. These completeness values are in alignment with the high-quality draft criterion of the Minimum Information about a Metagenome-Assembled Genome (MIMAG) standards for Bacteria and Archaea^19^ (Fig. 1, Table S2).

**Fig. 2:**
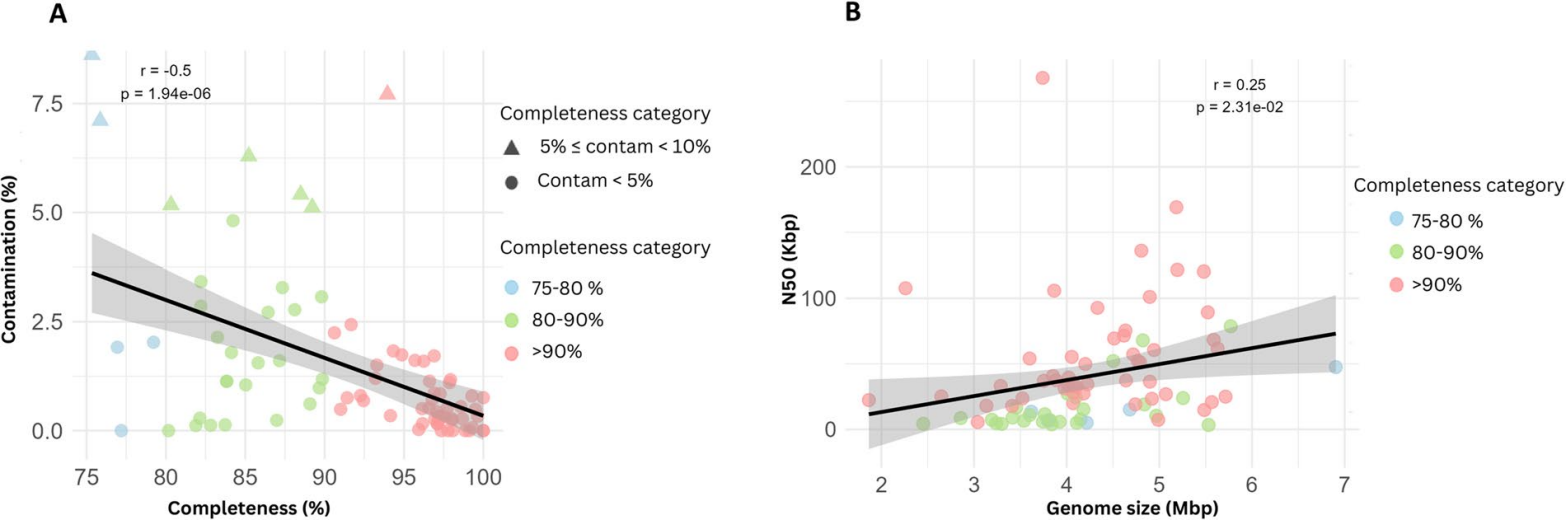
Correlations in Metagenome-Assembled Genomes (MAGs).

Furthermore, minimal levels of sequence heterogeneity were observed for all 82 MAGs. Approximately 91% (75/82) of the MAGs registered contamination levels <5%, whereas the remaining seven MAGS exhibited contaminant levels between 5 and 10%, ensuring the reliability and integrity of our dataset (Fig. 1 and Table S2). We identified a notable negative correlation between genome completeness and contamination (r = −0.498, p < 0.00001; Fig. 2A). In parallel, our data demonstrated a positive relationship between genome size and the N50 metric (r = 0.251, p = 0.023; Fig. 2B), indicating that larger genomes are often associated with superior assembly contiguity.

Taxonomic evaluation using the Genome Taxonomy Database Toolkit (GTDB-Tk)^20^ revealed that the barley-associated MAG dataset was dominated by members of the phylum Pseudomonadota (formerly the Proteobacteria), comprising 53.7% (44/82) of the total MAGs (Table S2) This is consistent with the findings from a previous amplicon sequencing-based study of barley seed endophytic microbial communities^7^. However, in contrast to the previous findings, we identified Bacteroidota (16/82) as the second most prevalent phylum. The abundances of Actinobacteria and Bacillota (Firmicutes) in our study also differed from those previously reported^7^, underscoring the inherent variability of barley seed microbiomes (Fig. 1 and Table S2).

### Temporal shifts in genera abundance over nine months

**Fig. 3:**
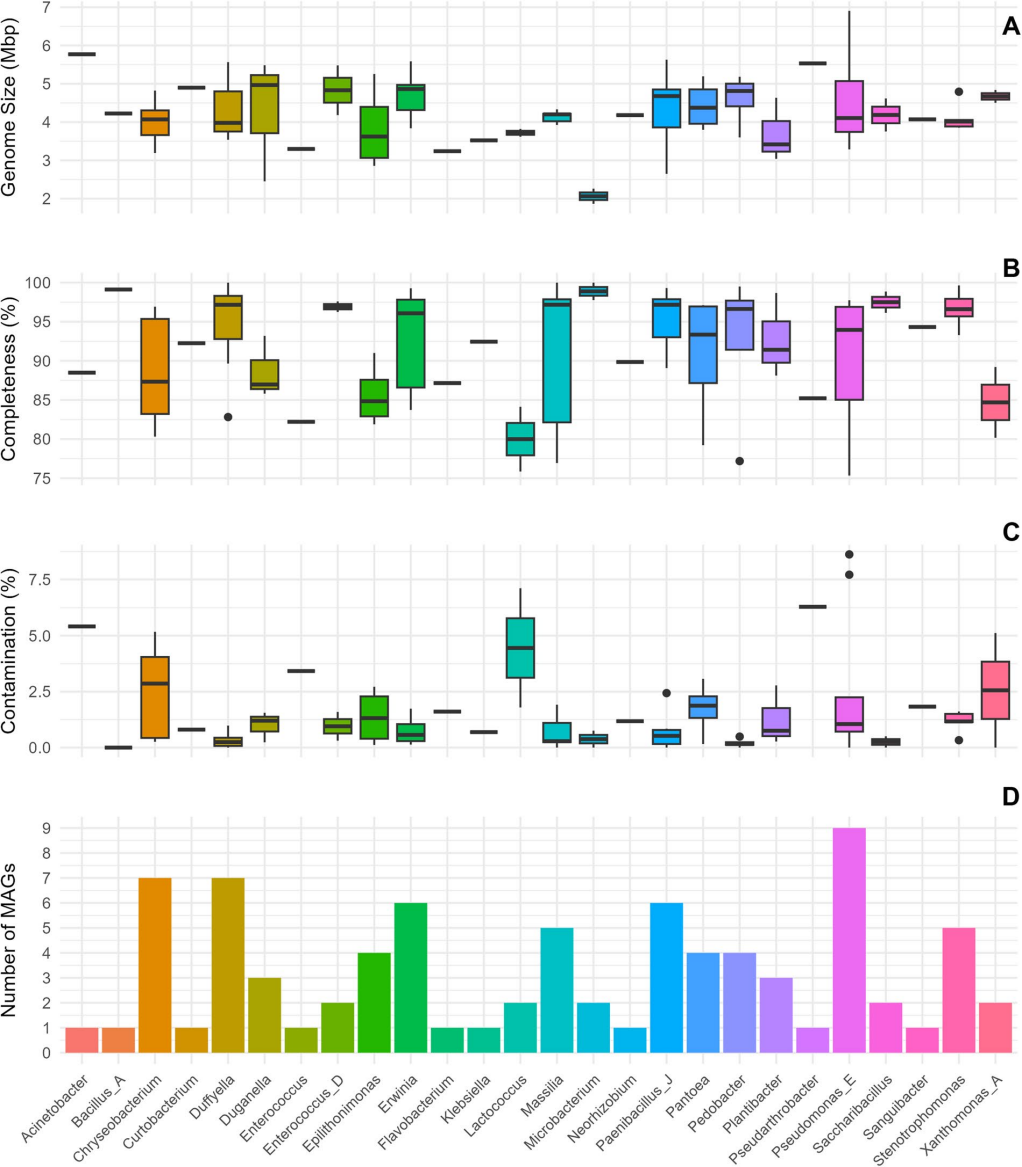
Genomic Metrics of the identified Bacterial Genera.

**Fig. 4:**
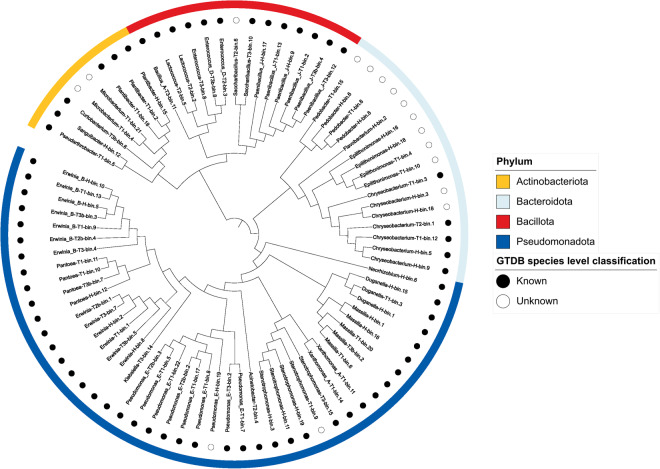
Phylogenetic Relationships of Bacterial MAGs.

The barley-seed derived MAGs were classified into 26 bacterial genera across eight phyla and six classes (Table S2). The microbiome was characterised by several dominant genera, with thirteen, nine, seven and six MAGs belonging to the genera *Erwinia*, *Pseudomonas*, *Chryseobacterium* and *Paenibacillus*, respectively (Fig. 3). Notably, 16 MAGs could not be accurately classified at the species level, highlighting the underexplored microbial diversity associated with barley seeds (Fig. 4, Table S2).

**Fig. 5:**
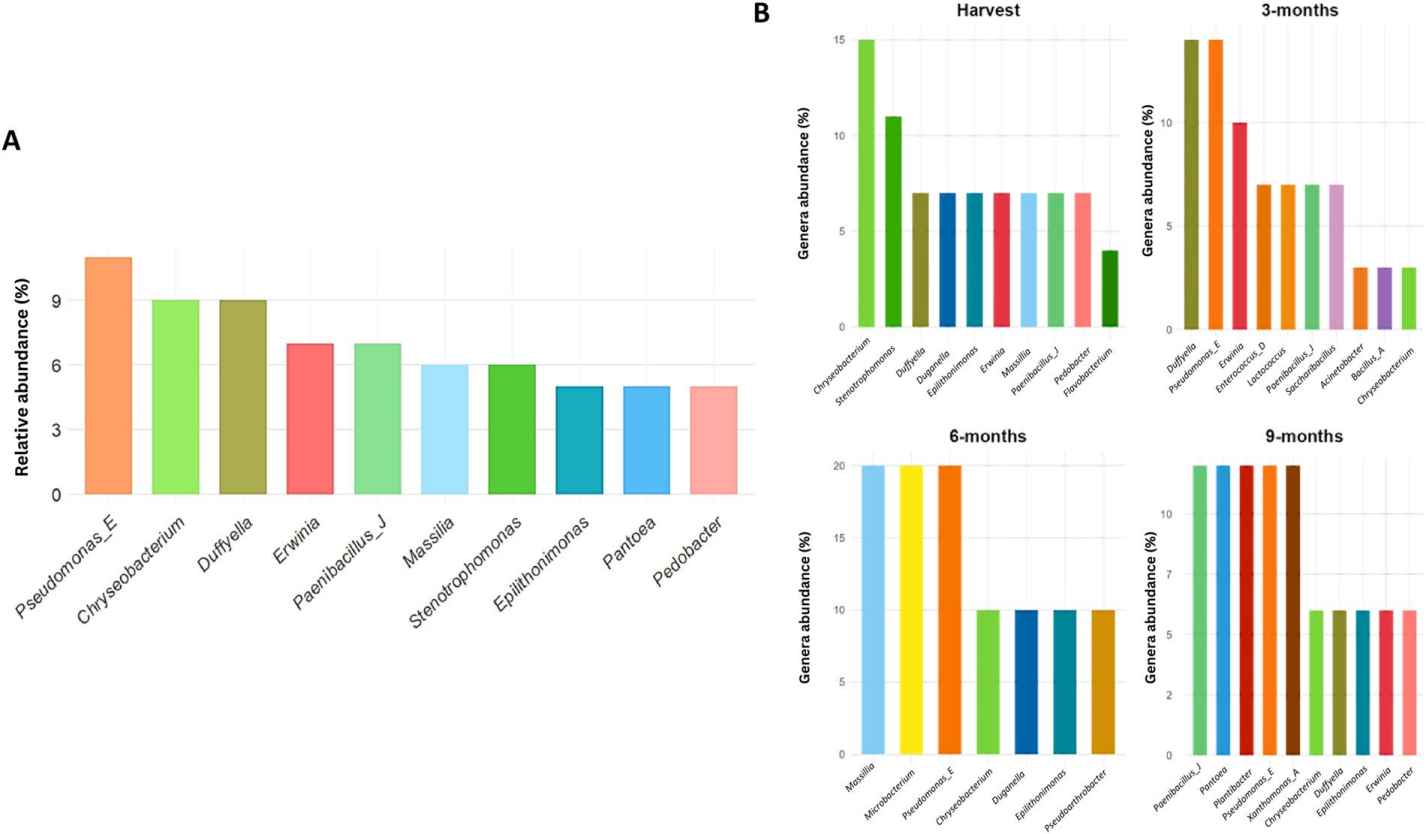
Combined plots illustrating the top 10 genera.

The barley seed microbiome shows discernible shifts during storage (Fig. 5). While the genera *Erwinia* and *Duffyella* remain pertinent from harvest through prolonged storage, there is a notable downshift and upshift in the presence of genera *Chryseobacterium* and *Pseudomonas*_E, respectively, during silo storage. These shifts may provide insights into the role of the barley seed microbiome in both seed health and disease. *Chryseobacterium* sp. have been observed to counteract the effects of *Magnaporthe oryzae*, a cause of barley blast disease, primarily by detaching fungal spores from leaf surfaces^21^, and may contribute to maintaining seed health in the field. *Duffyella* also garnered interest due to its observed ability to curb the growth of *Fusarium tricinctum*, another pathogen affecting barley^22,23^. All *Erwinia* MAGs identified in the study were classified in the species *E. persicina*, a known broad host range phytopathogen, which has been linked to pink seed disease in barley^24^. *Pseudomonas*-like taxa in this study were classified as part of the novel genus *Pseudomonas*_E as predicted by the GTDB classification database^20^.

## Methods

### Sample collection and processing

Malting barley (Hordeum *vulgare*) samples, of a single cultivar (Kadie), were sourced from Anheuser-Busch InBev (AB-Inbev) in South Africa., specifically from Storage facilities in the Western Cape province, South Africa, were selected. Samples were collected at four distinct time points: immediately post-harvest and then after three, six, and nine months of storage in silos. At each time point, three samples were collected. All samples were aseptically collected and stored at −20 °C to inhibit microbial growth.

### DNA isolation and sequencing

Approximately 10 g of barley was crushed using a sterilised mortar and pestle. The resulting residue was suspended in 40 ml of phosphate buffered saline (PBS) solution (pH 7.4). The suspension was briefly vortexed to homogenise the mixture, followed by sonication at 18 W amplitude with a 30-s on-off pulsating schedule for 7 min. The mixture was centrifuged at 4000 × g for 1 min to separate the supernatant, which was transferred to an autoclaved polycarbonate filter holder and filter membrane (0.45 µm pore filter, Sartorius-Stedim Biotech) prepared filter membrane system.

Metagenomic DNA was extracted from the filter using the ZymoBIOMICS DNA/RNA Miniprep Kit (Zymo Research), following the protocol recommended by the manufacturer. A Nanodrop Lite Spectrophotometer (Thermo Fisher Scientific) was used to validate the integrity and purity and quantify the DNA. The metagenomic DNA samples were sequenced using the Illumina NovaSeq. 6000 platform (paired end reads, 2 × 250 bp) at Molecular Research (MRDNA, Texas, USA). The total number of reads obtained was approximately 365.27 million. On average, each sample yielded around 22.83 million reads, with the maximum number of reads for a single sample being approximately 38.26 million and the minimum around 10.36 million. These metrics provide an overview of the sequencing depth achieved in our study. For a detailed breakdown of read counts for each sample (Table S1).

### Metagenomic data analysis

Raw sequence reads were evaluated for quality using FastQC v0.12.1^25^ and MultiQC v1.15^26^. Trimmomatic V0.36^27^ was used to filter out reads shorter than 36 bp or with an average quality score lower than 15. The removal of host DNA was performed using Bowtie2 v2.5.1^28^ and SAMtools v1.19^29^. Initially, an index database employing the reference genome of barley (*Hordeum vulgare*, Accession number: GCF_904849725.1) was constructed using the *bowtie2-build* command. Subsequently, read mapping to the host sequence database with Bowtie2 was conducted, preserving both aligned and unaligned paired end reads. Following this, SAMtools was used to convert the *sam* file into a *bam* format. The required unmapped reads were precisely isolated by applying SAMtools SAM-flag filters (-f 12 and -F 256), which selected pairs where both reads (R1 and R2) were unmapped. Finally, the *SAMtools sort* and *SAMtools fastq* commands were used to separate the paired end reads into distinct fastq files. Host DNA contamination varied across samples with the mean contamination ratio was approximately 0.5757%, with the minimum at 0.0059% (3,088 contaminated reads out of 52,678,404) and the maximum at 2.7368% (567,134 contaminated reads out of 20,155,530) (Table S1). Thereafter, the reads were then assembled using metaSPAdes v3.15.3^30^ with default parameters. The integrity and quality of the final assemblies were evaluated using QUAST v5.2.0^31^.

### Metagenomic binning and refinement

Metagenomic binning was performed based on tetranucleotide frequencies, coverage, and GC content using the MetaWRAP v1.3^32^ pipeline with default parameters using the tools MaxBin v2.0^33^, metaBAT2^34^, and CONCOCT v1.0.0^35^. The bins were refined further using the MetaWRAP-Bin_refinement module with the parameters -c 70 and -× 10 (completeness >70% and contamination <10%) to improve bin quality. The completeness and contamination levels of these genome segments were evaluated using CheckM v1.2.2^18^ as part of the MetaWRAP workflow. Subsequently, the bins were reassembled using the MetaWRAP-reassemble_bins module (parameters: -c 70 × 10). The refined bins were dereplicated at a 95% average nucleotide identity (ANI) threshold using dRep v2.6.2^36^, culminating in 82 nonredundant MAGs.

### Phylogenetic analysis and classification of MAGS

For taxonomic assignment of MAGs, the classify_wf workflow from GTDB-Tk v3.4.2^20^ was employed in tandem with the reference data GTDB release207v2^20^, all executed with default settings. A comprehensive phylogenetic tree encompassing 82 species-level bacterial MAGs was derived from 120 bacterial marker genes using the gtdbtk_infer module in GTDB-TK. To improve interpretation and visualisation, the tree was annotated using iTOL v5^37^.

## Data Records

The data records are available Figshare^38^.

The 82 MAGs have been deposited at DDBJ/ENA/GenBank under the accession numbers listed in Table 1^39–119^.

**Table 1:** Genomic characteristics and accession numbers of 82 microbial genomes from barley seed communities described in this study.

MAG name	Total length (Mb)	Contigs number	GC (%)	N50	Accession
MAG82-bin8	3,0	715	42.99	5647	GCA_037032585.1
MAG81-bin7	3,6	365	56.28	13890	GCA_037032605.1
MAG80-bin6	3,2	481	71.33	8733	GCA_037032625.1
MAG79-bin5	4,5	92	55.98	82812	GCA_037031965.1
MAG78-bin4	4,7	455	39.44	19164	GCA_037032645.1
MAG77-bin3	3,8	435	56.04	37421	GCA_037032685.1
MAG76-bin2	3,8	476	64.81	10259	GCA_037032705.1
MAG75-bin8	2,6	144	37.53	25727	GCA_037032665.1
MAG74-bin7	4,6	71	55.65	91004	GCA_037031985.1
MAG73-bin4	4,1	118	55.65	57133	GCA_037032725.1
MAG72-bin2	4,7	260	63.75	26823	GCA_037032745.1
MAG71-bin15	4,2	1366	67.28	19361	GCA_037032045.1
MAG70-bin14	5,5	92	56.06	113978	GCA_037032795.1
MAG69-bin12	3,7	912	39.59	6488	GCA_037032765.1
MAG68-bin11	4,1	809	34.99	5938	GCA_037032785.1
MAG67-bin10	5,0	833	59.53	8126	GCA_037032825.1
MAG66-bin4	4,0	142	55.43	51456	GCA_037032845.1
MAG65-bin3	5,1	263	61.48	29080	GCA_037032005.1
MAG64-bin2	5,3	310	59.62	25025	GCA_037032865.1
MAG63-bin1	4,5	72	55.87	96926	GCA_037032905.1
MAG62-bin6	5,5	1825	56.67	3460	GCA_037032925.1
MAG61-bin5	2,3	30	34.98	118370	GCA_037032025.1
MAG60-bin4	2,5	649	43.03	4356	GCA_037032945.1
MAG59-bin3	3,3	149	43.14	48287	GCA_037032885.1
MAG58-bin2	1,9	131	38.18	22371	GCA_037032965.1
MAG57-bin1	4,3	62	33.55	117162	GCA_037033005.1
MAG56-bin9	4,2	182	55.70	35876	GCA_037033045.1
MAG55-bin8	5,2	66	38.99	148394	GCA_037032985.1
MAG54-bin7	4,9	182	63.55	37031	GCA_037033025.1
MAG53-bin6	3,8	1068	65.23	4645	GCA_037033065.1
MAG52-bin5	5,6	134	60.65	64684	GCA_037033085.1
MAG51-bin4	3,7	126	38.87	267888	GCA_037033105.1
MAG50-bin3	4,1	281	39.58	49864	GCA_037033125.1
MAG49-bin2	3,3	840	69.80	4591	GCA_037033145.1
MAG48-bin13	4,9	281	39.48	27429	GCA_037033165.1
MAG47-bin12	3,5	584	34.27	7567	GCA_037033185.1
MAG46-bin11	3,7	742	68.29	6415	GCA_037033205.1
MAG45-bin10	4,6	387	55.28	59038	GCA_037033245.1
MAG44-bin9	3,5	204	66.62	25013	GCA_037033225.1
MAG43-bin8	4,8	326	59.56	101115	GCA_037033265.1
MAG42-bin5	4,2	161	66.34	38378	GCA_037033285.1
MAG41-bin4	3,8	152	67.87	37408	GCA_037033305.1
MAG40-bin3	5,6	263	64.22	31007	GCA_037033325.1
MAG39-bin22	5,4	928	61.45	25000	GCA_037033345.1
MAG38-bin21	3,4	278	69.06	17946	GCA_037033365.1
MAG37-bin20	4,2	380	64.71	14133	GCA_037033385.1
MAG36-bin2	4,6	148	39.64	80281	GCA_037033405.1
MAG35-bin18	3,6	331	69.26	13948	GCA_037033425.1
MAG34-bin17	5,6	446	59.19	85504	GCA_037033485.1
MAG33-bin15	5,4	175	39.01	122493	GCA_037033465.1
MAG32-bin14	3,8	599	68.39	8412	GCA_037033445.1
MAG31-bin13	4,1	167	55.76	35365	GCA_037033505.1
MAG30-bin11	3,8	394	56.22	13016	GCA_037033525.1
MAG29-bin10	3,5	195	39.11	54766	GCA_037033545.1
MAG28-bin1	4,8	135	55.97	64761	GCA_037033565.1
MAG27-bin9	4,7	150	39.64	57215	GCA_037033605.1
MAG26-bin8	4,9	142	39.06	74375	GCA_037033585.1
MAG25-bin6	4,2	304	60.69	23553	GCA_037033625.1
MAG24-bin5	3,9	88	34.12	108584	GCA_037033645.1
MAG23-bin3	3,5	250	34.81	22649	GCA_037033685.1
MAG22-bin2	4,8	99	55.63	152377	GCA_037033665.1
MAG21-bin19	6,9	396	61.22	51262	GCA_037033705.1
MAG20-bin18	2,9	404	39.91	10588	GCA_037033725.1
MAG19-bin16	3,9	583	64.95	8808	GCA_037033745.1
MAG18-bin15	3,4	296	70.06	17891	GCA_037033765.1
MAG17-bin12	4,1	262	70.83	20846	GCA_037033785.1
MAG16-bin11	4,1	88	65.80	72028	GCA_037033825.1
MAG15-bin10	4,0	137	55.75	51484	GCA_037033805.1
MAG14-bin1	5,5	473	64.32	17745	GCA_037033845.1
MAG13-bin9	4,0	354	34.00	25722	GCA_037033885.1
MAG12-bin8	5,6	150	54.51	92296	GCA_037033865.1
MAG11-bin6	5,1	137	38.97	193357	GCA_037033925.1
MAG10-bin5	4,1	121	55.66	62804	GCA_037033905.1
MAG9-bin3	3,7	365	65.94	42222	GCA_037033945.1
MAG8-bin2	3,2	862	39.87	4923	GCA_037034005.1
MAG7-bin19	3,7	448	66.41	43286	GCA_037033985.1
MAG6-bin18	4,7	451	34.43	73923	GCA_037034025.1
MAG5-bin17	4,6	242	39.66	37393	GCA_037033965.1
MAG4-bin16	3,1	197	39.55	25933	GCA_037034045.1
MAG3-bin15	5,0	511	64.14	11980	GCA_037034065.1
MAG2-bin12	3,9	428	55.44	30922	GCA_037034085.1
MAG1-bin1	4,2	618	64.66	9399	GCA_037034105.1

Additional metadata and details about each MAGs are available in the Supplementary Table S2.

The raw reads used to reconstruct the MAGs have been deposited to the NCBI Sequence Read Archive^120^.

## Technical Validation

Implementation of robust software applications, such as FastQC, MultiQC, and Trimmomatic, all of which were designed to curate and refine the sequence data. Combining the comprehensive MetaWRAP pipeline with dependable tools such as CheckM and GTDB-tk strengthened the binning, genome assembly, and taxonomic assignment processes. The culmination of these exhaustive validation stages is a dataset that is not only technically sound, but also a model of dependability and reproducibility in metagenomic research.

### Supplementary information

Table S1

Table S2

## Data Availability

No unique codes were used in the compilation or processing of this dataset. When applicable, the software versions and any deviations from default settings are explicitly indicated.
